# Outcome of Chronic Myeloid Leukemia Patients Not in Deep Molecular Response: Results From the GIMEMA LabNet CML Network Database

**DOI:** 10.1002/ajh.27733

**Published:** 2025-06-05

**Authors:** Fabio Stagno, Rosalba Cucci, Giovanni Marsili, Fausto Castagnetti, Sara Galimberti, Barbara Izzo, Federica Sorà, Simona Soverini, Monica Messina, Alfonso Piciocchi, Massimiliano Bonifacio, Daniela Cilloni, Alessandra Iurlo, Giovanni Martinelli, Gianantonio Rosti, Paola Fazi, Marco Vignetti, Massimo Breccia, Alessandro Allegra, Fabrizio Pane

**Affiliations:** ^1^ Hematology Section, AOU Policlinico “G. Martino” University of Messina Messina Italy; ^2^ GIMEMA Foundation Rome Italy; ^3^ Department of Medical and Surgical Sciences, Institute of Hematology “Seràgnoli” University of Bologna Bologna Italy; ^4^ Department of Clinical and Experimental Medicine, Hematology University of Pisa Pisa Italy; ^5^ Dipartimento di Mesdicina Molecolare e Biotecnologie Mediche Napoli Italy; ^6^ Institute of Hematology Policlinico Universitario A. Gemelli, “Cattolica” University Rome Italy; ^7^ Department of Engineering for Innovation Medicine, Section of Innovation Biomedicine, Hematology Area University of Verona Verona Italy; ^8^ SCDU Hematology and Cellular Therapies AO Ordine Mauriziano di Torino Torino Italy; ^9^ Hematology Foundation IRCCS Ca'Granda Ospedale Maggiore Policlinico Milan Italy; ^10^ IRCCS Istituto Romagnolo per lo Studio dei Tumori (IRST) “Dino Amadori” Meldola Italy; ^11^ Department of Translational and Precision Medicine, Az Policlinico Umberto I‐Sapienza University Rome Italy; ^12^ Division of Hematology and Bone Marrow Transplantation, Department of Medicine and Surgery University of Naples Federico II Naples Italy

To the Editor,

1

Chronic myeloid leukemia (CML) is currently managed as a chronic disease requiring long‐term treatment and a close molecular monitoring in many patients [1]. Evidence suggests that in a substantial number of patients who achieved a stable sustained deep molecular response (DMR) the treatment with tyrosine kinase inhibitors (TKIs) can be safely discontinued [2, 3]. Hence, treatment‐free remission (TFR) is a strategy increasingly considered as a feasible treatment goal in about 20%–40% of CML patients [4]. Nevertheless, a proportion of patients with CML in chronic‐phase (CP) treated with TKIs still remain in stable major molecular remission (MR3) or less (MR2 only) without achieving a DMR, therefore requiring long‐term TKIs therapy [5] as well as a long‐term molecular monitoring [6].

The Italian Group for Adult Hematological Malignancies (GIMEMA) activated in 2008 a project named GIMEMA LabNet CML network with the aim of reassuring a nationwide fast and harmonized molecular diagnostic and monitoring process to all CML patients. The network is now made up of 51 standardized laboratories for clinical and research purposes linked with 144 hematological centers. The connection between the hematology centers and laboratories is managed by a web‐based general data protection regulation (GDPR) compliant platform. LabNet digital infrastructure manages the traffic of molecular diagnostic tests between clinical centers and laboratories with the aim of providing a standardized evaluation of minimal residual disease. Therefore, if not all, most of the CML patients living in Italy are monitored for their disease at the same level of accuracy and harmonization.

The aim of our analysis was to describe, in the pure real‐life scenario of the GIMEMA LabNet CML network, the long‐term outcome of those CML patients in stable MR3/MR2. The LabNet CML database represented the data source of our analysis. To participate in the network, laboratories fulfilled quality controls and regularly participated in quality control rounds.

At the time of the current analysis, the LabNet database contained data of 9699 patients affected by CML with evaluable samples for *BCR::ABL1* transcripts. All patients gave written informed consent. We selected the patient cohort by analyzing all those with CP‐CML treated frontline with Imatinib (IM), Dasatinib (DAS), or Nilotinib (NIL) at conventional doses who achieved, within 6 months, an MR3 or less (MR2) and showed evaluable samples for molecular response at least 24 months from the first MR3 or MR2 achievement. We collected only *BCR::ABL1* kinetic and molecular data according to clinical practice. The LabNet CML Network adopted Subjective Objective Assessment and Plan (SOAP) notes according to the European LeukemiaNet guidelines [6]. The *BCR::ABL1* transcript levels were measured in each laboratory from peripheral blood samples drawn at diagnosis and then roughly every 3 months using real‐time PCR (qPCR) analysis as previously described [6]. ABL1 was used as the reference gene at any time point, and Real Time PCR (Q‐PCR) determinations for BCR::ABL1/ABL1 were converted to the international scale (IS).

Patient's characteristics were summarized by means of frequencies and percentage values for categorical variables, while continuous variables were described with median values and their relative ranges. The association between categorical variables was assessed using either Pearson's or Fisher's test, as appropriate. All tests were two‐sided with a significance level of 0.05, and confidence intervals were calculated at the 95% level. Since no data was available for a proper survival analysis, we used as a surrogate the observation time (OT) between the first MR3/MR2 response date and the date of the last sample collection. All analyses were performed using the R software version 4.2.2.

Five hundred eighty‐five out of 9699 patients included in the LabNet CML database met study endpoints and achieved a MR3 or MR2 within 6 months with a minimum follow‐up of 24 months (Figure 1). Particularly, 187 of them did not achieve a DMR and maintained a stable MR3/MR2 only. All patients were enrolled by 74 GIMEMA Centers across Italy. They all received conventional frontline therapy as follows: 219 with IM, 133 with DAS, and 233 with NIL. Median age at diagnosis was 58 years (IQR: 46–69), male was 55%, and female 45%, with a median OT of 5.5 years (range: 2–15). Within 24 months, 375 (64%) out of 585 patients achieved a DMR (MR4, MR4,5), whereas 187 showed a stable MR3/MR2 response and 23 had an unstable MR2 showing also some molecular fluctuations less than MR2 (Table 1). No difference in achieving a MR equal to or better than MR3 was observed using the three different TKIs frontline (*p* > 0.9) as well as among different age groups (18–45; 45–65; > 65; *p* = 0.3). A trend toward greater likelihood to achieve DMR was observed for those CML patients carrying the e14a2 transcript type (*p* = 0.02). Hence, we focused our attention on the stable MR3/MR2 cohort (187 pts) in the subsequent > 24 months of follow‐up (Table 2). With the successive follow‐up, 123 out of 187 (66%) achieved a DMR, 59/187 (32%) remained in stable molecular response, and 5/187 (2%) lost the response. The three TKIs elicited comparable rates of MR (*p* = 0.2) and no difference was observed among different age groups (18–45; 45–65; > 65; *p* = 0.7). The median OT was 5.5 years for the total study group (585 pts), 5.5 years for the DMR group (375 pts), 5.4 years for the stable MR2/MR3 (187 pts) and 4.8 years for the unstable MR2 ones (23 pts).

**FIGURE 1:**
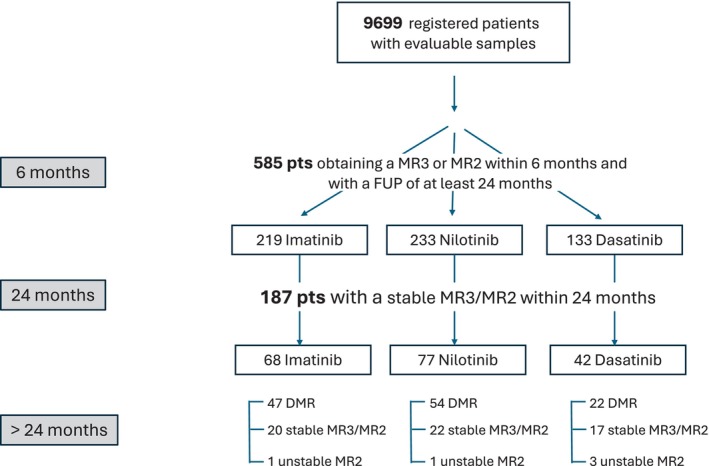
Consort diagram of the total CML population.

**TABLE 1:** Molecular response: Initial follow‐up at 24 months.

	*N*	Overall, *N* = 585	Imatinib, *N* = 219	Dasatinib, *N* = 133	Nilotinib, *N* = 233	Median observation time, years (range)
24 months, *n* (%)	585					5.5 (2.01–15.08)
DMR (MR4, MR4.5)		375 (64%)	143 (65%)	84 (63%)	148 (64%)	5.56 (2.01–11.69)
Stable MR3/MR2		187 (32%)	68 (31%)	42 (32%)	77 (33%)	5.48 (2.03–15.08)
Unstable MR2		23 (4%)	8 (4%)	7 (5%)	8 (3%)	4.83 (2.41–10.23)

**TABLE 2:** Molecular behavior of the stable MR3/MR2 cohort (187 pts): Second follow‐up.

Two follow up	*N*	Overall, *N* = 187	Imatinib, *N* = 68	Dasatinib, *N* = 42	Nilotinib, *N* = 77	Median observation time, years (range)
*n* (%)	187					5.48 (2.03–15.08)
DMR		123 (66%)	47 (69%)	22 (52%)	54 (70%)	6.38 (2.48–15.08)
Stable MR3/MR2		59 (32%)	20 (29%)	17 (40%)	22 (29%)	3.51 (2.03–10.69)
Unstable MR2		5 (2%)	1 (2%)	3 (8%)	1 (1%)	5.79 (2.67–9.14)

We then compared the unstable MR2 cohort (23 patients) in the initial 24 months follow‐up with MR2/MR3 and DMR patients. As expected, we found a lower rate of e14a2 transcript in both the stable and unstable MR2 cohorts (41%, *p* = 0.021). In this setting, molecular *BCR::ABL1* fluctuations within 48 months predicted the loss of MR2.

The GIMEMA LabNet CML network represents in Europe a unique model of harmonization and standardization of molecular biology laboratories dedicated to CML. We are aware that many clinical and prognostic data are missing because of LabNet's scope, but our intention was to focus on the molecular data in a large cohort of patients and in a real‐life scenario.

Results obtained on 585 CP‐CML patients treated frontline with TKI therapy showed that a MR3/MR2 stable response achieved within 24 months is predictive, as expected by previous evidence [7, 8, 9], of a good molecular response since 66% of these patients gained a DMR subsequently. On the other side, 32% of the patients with CML remained in stable MR3/MR2 response. In this view, no TKI switch should be offered for patients in stable MR3/MR2 at 24 months since clinical evidence shows that a proportion of them will achieve later a DMR. Unfortunately, others will remain in stable MR3 and will require lifelong TKI therapy [5]. Therefore, therapeutic strategies are needed in this clinical setting to optimize treatment either in a pro‐active or in a conservative way. Conversely, those patients showing molecular fluctuations need to be carefully monitored to prevent progression and may benefit from treatment with alternative TKIs [10, 11, 12, 13]. In conclusion, these findings obtained through the GIMEMA LabNet CML network and in a large series of CML patients in Italy, depict the real‐life molecular scenario and outcome of those patients with CML not in DMR and witness the power of the GIMEMA project that can be further implemented with clinical data. The current analysis represents a pilot investigation since we are planning further and larger analyses in the future based on roughly 10 000 patients molecularly monitored during a long period of time.

## Author Contributions

Fa.St., R.C., G.M., F.C., S.G., B.I., Fe.So., S.S., M.M., A.P., M.B., D.C., A.I., G.M., G.R., P.F., M.V., M.B., and F.P. designed the study. R.C., G.M., M.M., and A.P. provided and/or collected the data. Fa.St. and G.R. wrote the paper. Fa.St., G.R., M.B., A.I., M.M., M.B., M.V., A.A., and F.P. reviewed the paper.

## Ethics Statement

This study was conducted in accordance with the Declaration of Helsinki, and clinical management was performed according to current guidelines.

## Consent

Written informed consent was obtained from the patients.

## Conflicts of Interest

F.S.: Incyte, Novartis, Janssen; F.C.: Novartis, Incyte; S.G.: Roche, Incyte, Novartis, Jazz, AstraZeneca, AbbVie, Celgene, Pfizer, Janssen; F.S.: Novartis; S.S.: Blueprint Medicines, Incyte, Istituto Gentili; M.B.: Pfizer, Amgen, Incyte, Novartis, BMS; G.M.: Novartis, BMS, ARIAD, MSD, Roche, Pfizer; G.R.: Pfizer, Novartis, Incyte; M.V.: Mattioli Health, Arhea, Edrea, Vertex, Isheo, Novartis, Abbvie, AstraZeneca, Dephaforum SRL; M.B.: GSK, BMS, Abbvie, AOP, Incyte, Pfizer, Novartis; F.P.: GSK, Incyte, Amgen, BMS, Janssen, Jazz, Novartis, Pfizer, GSK, Incyte. The other authors declare no conflicts of interest.

## Data Availability

The data supporting the findings described in this study are available from both the corresponding author and the GIMEMA LabNet CML Network upon reasonable request.
